# The risk to import ESBL-producing Enterobacteriaceae and *Staphylococcus aureus* through chicken meat trade in Gabon

**DOI:** 10.1186/s12866-014-0286-3

**Published:** 2014-11-19

**Authors:** Frieder Schaumburg, Abraham S Alabi, Lisa Frielinghaus, Martin P Grobusch, Robin Köck, Karsten Becker, Saadou Issifou, Peter G Kremsner, Georg Peters, Alexander Mellmann

**Affiliations:** Institute of Medical Microbiology, University Hospital Münster, Domagkstr. 10, 48149 Münster, Germany; Centre de Recherches Médicales de Lambaréné, Albert Schweitzer Hospital, Lambaréné, Gabon; Institut für Tropenmedizin, Eberhard Karls Universität and Deutsches Zentrum für Infektionsforschung, Tübingen, Deutschland; Department of Infectious Diseases, Division of Internal Medicine, Center for Tropical Medicine and Travel Medicine, Academic Medical Center, University of Amsterdam, Amsterdam, The Netherlands; Institute of Hygiene, University Hospital Münster, Münster, Germany

**Keywords:** Staphylococcus aureus, Escherichia coli, Antimicrobial resistance, Chicken meat, Trade, Africa

## Abstract

**Background:**

A main export market for chicken meat from industrialized countries is sub-Saharan Africa. We hypothesized that antibiotic resistant bacteria could be exported to developing countries through chicken meat trade. The objective was to investigate the occurrence and molecular types of ESBL-producing Enterobacteriaceae and *Staphylococcus aureus* in chicken meat in Gabon and to assess their dissemination among humans.

**Results:**

Frozen chicken meat samples imported from industrialized countries to Gabon (n = 151) were screened for ESBL-producing Enterobacteriaceae and *S. aureus*. Genotypes and resistance genes (SHV, TEM, CTX-M, CMY-2) of isolates from meat were compared with isolates derived from humans.

The contamination rate per chicken part (i. e. leg, wing) with ESBL-producing *Escherichia coli* (ESBL *E. coli*, no other ESBL-producing Enterobacteriaceae were found) and *S. aureus* was 23% and 3%, respectively. The beta-lactamase CTX-M 1 was predominant in ESBL *E. coli* from meat samples but was not found in isolates from cases of human colonization or infection. *S. aureus* belonging to *spa* type t002 (multilocus sequence type ST5) were found both in chicken meat and humans.

**Conclusion:**

There is a risk to import ESBL *E. coli* to Gabon but molecular differences between isolates from humans and chicken meat argue against a further dissemination. No MRSA isolate was detected in imported chicken meat.

## Background

The global trade volume is steadily increasing and the exchange of various goods may contribute to economic wealth and development. However, global travel and trade bear the risk of transmitting pathogens through travelers or goods. While measles, syphilis, tuberculosis or smallpox have spread in the age of exploration and colonization, nowadays, new pathogens are emerging [1].

For instance, enterohemorrhagic *Escherichia coli* O104:H4, which caused an outbreak in central Europe in 2011, was most likely imported through seeds from Egypt [2]. Similarly, international travel, transfer of patients and medical tourists facilitate the import of various pathogens such as extended spectrum beta-lactamase (ESBL)- and carbapenemase-producing Enterobacteriaceae or Panton-Valentine leukocidin (PVL) positive *Staphylococcus aureus* [3–5].

The frequent use of antibiotics - not only as therapeutics but also as growth promoters - has contributed to the emergence of antibiotic resistance in animal husbandry [6]. Hence, in European countries, industrially raised poultry and chicken can be contaminated with ESBL-producing *E. coli* (ESBL *E. coli*, up to 58–93.3% of chicken) and methicillin-resistant *S. aureus* (MRSA, 0.7-37.2% of chicken) [7–12]. One study showed that contamination of food items with multidrug-resistant bacteria might be an important vehicle for the spread of antibiotic resistance [13].

While current literature mostly indicates an import of antimicrobial resistant pathogens from developing countries to the industrialized world, there is a potential transmission pathway for such bacteria, which is diametrically opposed: Many industrialized countries do not only produce poultry meat for their domestic market but also for export to low income or developing countries. The leading exporters of poultry meat in 2007 were the USA, Brazil and the Netherlands. Main import markets were sub-Saharan Africa and Asia (http://kids.fao.org/glipha).

Our hypothesis was that the import of meat from industrialized countries contributes to the emergence of multidrug resistance among humans in developing countries such as Gabon, Central Africa [14]. We therefore analyzed the contamination of chicken meat with ESBL-producing Enterobacteriacae (ESBL Enterobacteriaceae) and *S. aureus* in Gabon and compared genotypes of these pathogens with isolates from human carriage and infection.

## Results

### Origin of samples

**Figure 1:**
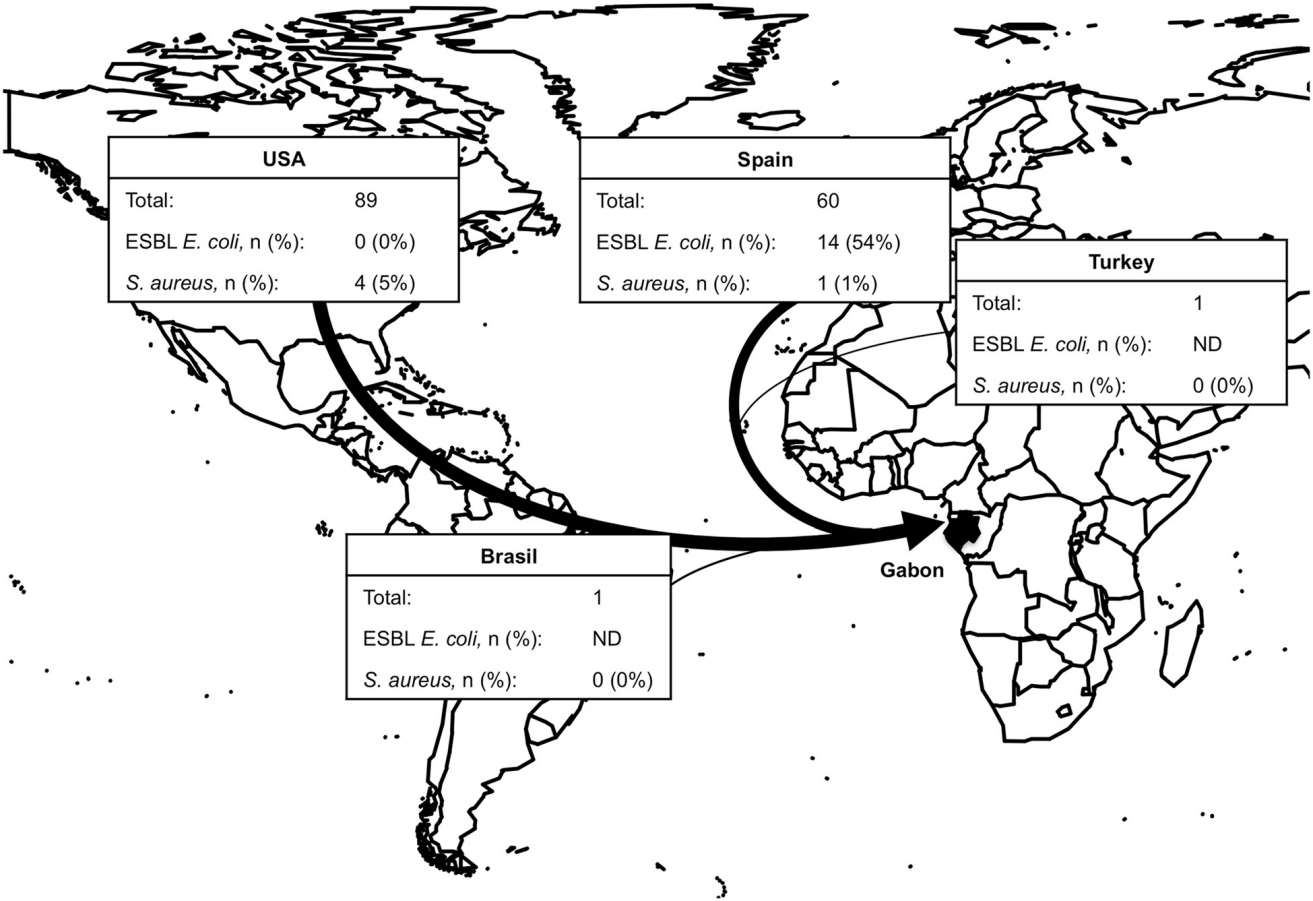
**Origin of imported poultry meat in Gabon.** The total number of samples and the proportion of ESBL *E. coli* and *S. aureus* in each country are shown. Screening for ESBL-producing Enterobacteriaceae was only done for a subset of samples from the USA (n = 34) and Spain (n = 26). Width of arrows represents the amount of imported poultry. The map was created with “R” (package “maps”).

In total, 151 chicken meat samples (88 legs, 63 wings) were analyzed. The chicken samples were frozen during shipment and storage in the supermarkets. Chicken meat was imported from the USA (n = 89), Spain (n = 60), Brazil and Turkey (each n = 1, Figure 1). No meat from domestic markets was sold in the five studied supermarkets in Lambaréné, Gabon.

### ESBL Enterobacteriaceae

The prevalence of ESBL *E. coli* in chicken meat was 23% (n = 14 of 60, Table 1). ESBL Enterobacteriaceae other than *E. coli* were not detected on the colorimetric media. We therefore included only ESBL *E. coli* isolates from human carriage and infection for comparison. Participants provided an informed consent prior to sampling. Isolates from humans came from already existing strain collections (Table 2). Among imported chicken meat in Gabon, ESBL *E. coli* was clearly associated with meat samples from Spain as the producing country (OR = Inf., 95% CI: 7.5–Inf., p < 0.001). The phylogenetic groups A and B1 were predominant in ESBL *E. coli* from chicken meat. In contrast, the other *E. coli* phylogenetic groups were more equally distributed among isolates from humans (Table 3). The beta-lactamases CTX-M 1 and CTX-M 14 were predominant in ESBL *E. coli* from chicken meat. However, these beta-lactamases were not found in isolates from humans. In contrast, the predominant beta-lactamase in isolates from human carriage and infection was CTX-M 15 (Table 3). Other beta-lactamases (TEM, SHV) were also found in ESBL *E. coli* from chicken meat or human carriage and infection. The plasmid-mediated AmpC beta-lactamase CMY-2 was not detected among ESBL *E. coli* isolates.

**Table 1:** **Description of poultry samples from Gabon**

	**No. (%)**
Imported chicken meat samples	151 (100%)
Samples screened for *S. aureus*	151 (100%)
Samples screened for ESBL *E. coli* ^a^	60 (39.7%)
ESBL *E. coli*	14 (23.3%)^b^
*S. aureus*	5 (3.3%)

^a^The consecutive screening for ESBL-producing Enterobacteria was started after the beginning of the study. Therefore not all meat samples were screened for ESBL-producing Enterobacteriaceae.

^b^Three samples were contaminated with two different *E. coli* isolates. These isolates were phenotypically different and harbored different beta-lactamase encoding genes.

**Table 2:** **Sources of ESBL** ***E. coli*** **and** ***S. aureus*** **isolates from human carriage and infection**

**Characteristics**	**ESBL** ***E. coli***		***S. aureus***	
	**Carriage (n = 16)**	**Infection (n = 10)**	**Carriage (n = 20)**	**Infection (n = 21)**
Year of collection	2010-2011	2010-2013	2011	2012
Median age (range)	1.5 (0.8- 12.5)	3.1 (0.2-68.8)	24.5 (6.5-37.3)	1.6 (0.2-36.2)
Females, n (%)	7 (43.8%)	4 (44.4%)	11 (55%)	9 (42.9)
Type of samples (n)	Rectal swabs (16)	Wound swabs (3), urine (2), others (5)	Nasal swabs (20)	Abscess (9), skin and soft tissue infection (12)
Reference	[14]	Routine diagnostics	[31]	Routine diagnostics

**Table 3:** **Characteristics of ESBL** ***E. coli*** **from poultry, colonization and infection in humans in Gabon**

		**Chicken meat (n = 17)**	**Carriage (n = 16)**	**Infection (n = 10)**
Phylogenetic group	A	13 (76.5%)	9 (56.3%)	2 (20%)
	B1	3 (17.7%)	4 (25.0%)	3 (30%)
	B2	-	2 (12.5%)	3 (30%)
	D	1 (5.9%)	1 (6.3%)	2 (20%)
Beta lactamases	CTX-M 1	4 (23.5%)	-	-
	CTX-M 14	6 (35.3%)	-	-
	CTX-M 15	-	16 (100%)	9 (90%)
	CTX-M 27	-	-	1 (10%)
	CTX-M 32	1 (5.9%)	-	-
	TEM	6 (35.3%)	7 (43.8%)	3 (30%)
	SHV	7 (41.2%)	-	1 (10%)
Antibiotic resistance, tested substances	Piperacillin/Tazobactam	1 (5.9%)	5 (31.2%)	1 (10%)
	Cefuroxim	15 (88.2%)	16 (100%)	10 (100%)
	Cefotaxim	12 (70.6%)	16 (100%)	10 (100%)
	Ceftazidim	7 (41.2%)	14 (87.5%)	10 (100%)
	Gentamicin	6 (35.3%)	5 (31.2%)	4 (40%)
	Ciprofloxacin	8 (47.1%)	9 (56.2%)	8 (80%)
	Cotrimoxazole	3 (17.6%)	15 (93.8%)	10 (100%)

Note: all figures are no (%), “-“ denotes 0 (0%).

The antibiotic resistance rates were lower in ESBL *E. coli* from chicken meat, compared to isolates from human carriage and infection (Table 3). The highest non-beta-lactam resistance in chicken meat was against ciprofloxacin (n = 8). No resistance was detected against meropenem, imipenem or ertapenem.

#### Staphylococcus aureus

In total, 3% (n = 5) of all meat samples were contaminated with *S. aureus* in Gabon. No MRSA was detected as confirmed by the absence of *mecA*.

*S. aureus* was more frequently found in poultry meat from the USA (5%) compared to Spain (1%, Figure 1). Isolates from chicken meat mainly belonged to multilocus sequence type (MLST) clonal complex (CC) CC5 which was associated with sequence type ST5 and *spa* types t002 (n = 3). Three so far unknown STs were detected and assigned to ST2622 (chicken, t386), ST2666 (human carriage, t084), ST2702 (human infection, t272, Table 4). Due to repeated amplification failure, we were unable to determine the ST for one isolates belonging to *spa* type t591 (chicken, MLST profile: ?-1-1-1-1-1-1, CC5).

**Table 4:** **Comparison of** ***spa*** **types and MLST STs of** ***S. aureus*** **from poultry, carriage and infection in humans in Gabon**

**Clonal complex**	**Sequence type**	***Spa*** **type (n)**		
		**Chicken meat (n = 5)**	**Carriage (n = 20)**	**Infection (n = 21)**
CC5	ST5	t002 (3)	t002 (1), t045 (1), t653 (1)	t319 (1)
	ST8	-	t008 (1)	t1476 (2), t121 (1)
	ST9	-	t1045 (1)	-
	ST15	-	t7583 (1)	t084 (6)
	ST25	-	-	-
	ST72	-	t9147 (1)	-
	ST97	-	t311 (1)	-
	ST2666	-	t084 (2)	-
	Non typable	t591 (1)	-	-
CC45	ST45	-	t939 (3), t6552 (2)	-
	ST508	-	t9149 (1), t2194 (2)	-
CC121	ST2702	-	-	t272 (1)
CC152	ST152	-	t355 (1), t454 (1)	t355 (9)
CC398	ST398	-	-	-
CC707	ST707	-	-	-
Singletons	ST1193	-	-	346 (1)
	ST2622	t386 (1)	-	-

Note: not detected (−).

Isolates belonging to t002 (ST5) were found in chicken meat and humans (Table 4). Of note, resistance rates for penicillin were always lower in isolates from chicken meat compared to isolates from human carriage and infection (Table 5).

**Table 5:** **Antimicrobial resistance of** ***S. aureus*** **from chicken meat, carriage and infection in humans in Gabon**

	**Chicken meat (n = 5)**	**Carriage (n = 20)**	**Infection (n = 21)**
Penicillin	1 (20%)	20 (100%)	21 (100%)
Oxacillin	0 (0%)	1 (5%)	1 (4.8%)
Gentamicin	0 (0%)	1 (5%)	0 (0%)
Erythromycin	0 (0%)	4 (20%)	1 (4.8%)
Clindamycin	0 (0%)	0 (0%)	0 (0%)
Tetracycline	2 (40%)	5 (25%)	14 (66.7%)
Cotrimoxazole	0 (0%)	4 (20%)	12 (57.1%)

Note: all figures are no (%).

## Discussion

We assessed the import and potential spread of ESBL *E. coli* and *S. aureus* through chicken meat in Gabon, Central Africa. Main findings were a high proportion of imported chicken meat in our sample collection and a high prevalence of ESBL *E. coli*. Beta-lactamases associated with chicken meat were not found in ESBL *E. coli* from carriage and infection in humans. Chicken-related *S. aureus* genotypes were found in human isolates from carriage and infection.

The high proportion of imported chicken products in Gabon is not surprising because the country has an excess of import of poultry meat. The import of poultry products increased in Gabon from 11,149 Mt (1997) to 43,875 Mt (2007), the ratio of exports to imports in 2007 was 0.0 (Germany: 0.8, http://kids.fao.org/glipha).

No ESBL Enterobacteriaceae other than ESBL *E. coli* were detected which is in line with recent reports where the proportion of ESBL *E. coli* among all ESBL-producers from chicken was 93.8% (Germany), 76.8% (the Netherlands) and 58-67% (Spain) [8,11,15]. The prevalence of ESBL *E. coli* in chicken meat was lower in chicken products from the US compared to Spain which could be explained by a generally lower ESBL *E. coli* contamination rate in chicken from the US [11]. This lower contamination rate might be achieved by a “pathogen reduction treatment” with chemical compounds (e. g. chlorinated water) in the US which is prohibited in the European Union [16].

The predominance of the phylogenetic groups A and B1 in poultry products was expected as these groups are mostly found in the environment in contrast to B2 which is associated with mammals [17].

The ESBL encoding genes found in chicken meat in Gabon mainly belonged to CTX-M 1 and CTX-M 14 (Table 3). The predominance of CTX-M 14 over CTX-M 1 reflects the proportion of CTX-M subtypes in Spain [7]. Our data do not provide any evidence for a transmission of ESBL *E. coli* from poultry to humans as CTX-M 1 and CTX-M 14 from chicken meat were neither detected in ESBL *E. coli* from carriage nor infection in humans (Table 3). This is in contrast to Europe and the Americas, where CTX-M 1 was the most common beta-lactamase in ESBL *E. coli* from humans [18].

It is unclear why the same ESBL subtype (CTX-M 1) occur in poultry related and human isolates in Europe but not in Gabon [18]. One might argue that the prevalence and concentration of ESBL *E. coli* in chicken meat in Gabon is not yet high enough to be transmitted and to establish a sustainable colonization in humans [19]. It is also possible, that our samples size was too small to detect CTX-M subtypes with lower prevalence such as CTX-M 1 which accounts for 7% of all ESBL/AmpC types in humans in Europe [18].

Chicken meat can be contaminated not only with ESBL Enterobacteriaceae but also with *S. aureus* and MRSA, in particular. *S. aureus* research in livestock animals has been focused on MRSA. The prevalence of MRSA in poultry products is up to 37.2% in Germany [10,20], but varies significantly within and between the different countries in Europe (0% in Switzerland, 6.9% in the Netherlands) [10,12]. This variation in MRSA colonization might be the reason why we did not detect any MRSA among the *S. aureus* isolates in our study. Nevertheless, genotyping of methicillin susceptible *S. aureus* (MSSA) revealed that some *S. aureus* from poultry meat and humans in Gabon share the same *spa* type (t002, ST5). On the one hand, it was postulated that a human-to-poultry transmission of *S. aureus* ST5 isolates occurred in the 1980s and that this clonal lineage now spreads in chicken around the world (except for Australia, where stringent controls of imported poultry products are applied) [21]. On the other hand, there might be a risk of transmission of isolates belonging to t002 back to humans [22].

Although our study provides important information about the spread of pathogens through the trade with chicken products, some limitations need to be addressed. First, the prevalence of *S. aureus* in chicken meat in Gabon might have been underestimated as we did not use *S. aureus* selective enrichment broths and chromogenic agars. This practice was chosen because our objective was to use preferentially methods which can be easily applied in rather basally equipped laboratories. Second, few months after the start of the study we realized the high burden of ESBL-producing Enterobacteriaceae in humans Gabon [14]. From that point, we decided to use chromogenic media for the screening of ESBL *E. coli* in chicken meat. Therefore only a proportion of samples were consecutively screened for ESBL-producing Enterobacteriaceae (60 of 151, Table 1). Third, more comprehensive genotyping approaches (e.g. sequencing of plasmids) would have been preferred to detect more reliably potential sources of transmission. Fourth, our results might not be representative for the whole country due to the small sample size. Fifth, due to the cross sectional study design we cannot draw any conclusions regarding the direction of transmission between chicken meat and humans, in case there is any. Sixth, the chromogenic agar that we used might suppress the growth of Enterobacteriaceae carrying the AmpC resistance type. Therefore, the prevalence of CMY-2 refers to ESBL-producers only.

## Conclusions

In conclusion, there is a risk to import ESBL *E. coli* to Gabon but molecular differences between isolates from humans and chicken meat argue against a further dissemination. No MRSA isolate was imported through poultry meat.

## Methods

### Ethical clearance

No ethical approval was sought to analyze chicken meat samples. In Gabon, no ethical approval is requested if already existing isolates from clinical samples are analysed [23]. Ethical clearance was obtained from our institutional review board (IRB) in Lambaréné to collect swabs from asymptomatic healthy carriers (Comité d’Éthique Régional Indépendant de Lambaréné, CERIL). The ESBL Enterobacteriaceae from carriers derived from children and the legal representative of each child gave documented oral informed consent prior to enrolment [14]. The IRB approved the use of documented oral informed consent to collect rectal swabs from children (CERIL 09/2010). Written informed consent was given to screen for nasal colonization with *S. aureus*. The use of written informed consent to obtain nasal swabs was approved by our IRB (CERIL 15/2009).

### Sample collection

In a cross sectional study, samples from frozen chicken meat (wing, leg) sold at five commercial markets in Lambaréné, Gabon were randomly collected from December 2011 to November 2012. All samples were originally designated for human consumption and were stored in freezers until sampling. The country of origin and the species (i.e. chicken) were recorded according to declarations on the packaging. To guarantee sterile sampling conditions, approximately 5 g of meat were sampled using gloves, sterile forceps and scalpels. Only one sample per selling unit/batch was taken. Samples were stored at −20°C until microbiological culture.

Our initial objective was to investigate the occurrence of *S. aureus* in chicken meat, but since a recent study showed a high carriage rate of ESBL Enterobacteriaceae in Gabonese children, we also included the screening for ESBL Enterobacteriaceae after beginning of the study (Table 1) [14].

The poultry-related isolates were compared with ESBL Enterobacteriaceae (matched for species found in chicken meat samples) and *S. aureus* from humans (carriage and infection) from already existing strain collections (Table 2).

Human isolates from infection derived from routine diagnostics of the Albert Schweitzer Hospital, Lambaréné, Gabon and were consecutively collected.

### Detection of ESBL-producing Enterobacteriaceae

The meat samples were directly streaked on colorimetric ESBL-selective agar plates (bioMérieux, Marcy l’Etoile, France). After overnight culture in non-selective brain-heart-infusion enrichment broth, the samples were again plated on ESBL-selective agar plates. Colorimetric colonies on ESBL-plates were subjected to species identification and susceptibility testing using Vitek 2 automated systems (bioMérieux).

Antimicrobial resistance was evaluated using EUCAST breakpoints for susceptibility testing (Version 3.1, 2011); susceptibility of ESBL-producing Enterobacteriaceae to beta-lactams was reported as found. We confirmed the ESBL phenotype by double-disc diffusion tests according to the manufacturer’s instruction (Mast discs, Mast diagnostics, Bootle, UK). A PCR was used to detect the genes encoding the beta-lactamases *bla*_SHV_, *bla*_TEM_, *bla*_CTX-M_ and *bla*_CMY-2_; the *bla*_CTX-M_ genes were sequenced for subtyping [24,25].

### Detection of *Staphylococcus aureus*

The meat samples were directly streaked on Columbia blood agar plates supplemented with aztreonam disks (13 μg; Oxoid, Hants, UK). After overnight culture in non-selective brain-heart-infusion enrichment broth, the samples were again plated on Columbia blood agar (supplemented with aztreonam disks). *S. aureus* was identified by standard procedures including an agglutination test (Pastorex Staph-Plus; Bio-Rad Laboratories, Marnes-la-Coquette, France). Presumptive *S. aureus* colonies were subjected to species identification and susceptibility testing using Vitek 2 automated systems (bioMérieux). Species confirmation was done by the detection of *nuc* [26].

Antimicrobial resistance was evaluated using EUCAST breakpoints for susceptibility testing (Version 3.1, 2011); Resistance to penicillin and oxacillin were confirmed by the detection of *blaZ* and *mecA*, respectively [26,27].

### Genotyping

Each ESBL *E. coli* isolate was assigned to its phylogenetic group (A, B1, B2, D) according to the genetic pattern of *chuA*, *yjaA* and the anonymous DNA Fragment TSPE4.C2 [28]. *S. aureus* was *spa* typed and multilocus sequence typing (MLST) was done exemplarily for one isolate of each *spa* type for each group (chicken, carriage and infection in humans) [29–31]. MLST clonal complexes (CC) were determined with eBURST using the whole MLST dataset and the stringent group definition of six of seven shared alleles (http://saureus.mlst.net/eburst).

### Statistics

Associations between categorical variables were tested using Fisher’s exact test. The odds ratio (OR) and 95% confidence interval (95%CI) were calculated to assess the strength of association. The significance level was set at 5%. Statistical analyses were performed using “R” (http://cran.r-project.org) and the package “Epicalc”. The map was created with “R” and the package “Maps”.

## Abbreviations

CC: Clonal complex
ESBL: Extended-spectrum beta-lactamase
MLST: Multilocus sequence typing
MRSA: Methicillin-resistant *Staphylococcus aureus*
ST: Sequence type
